# Preparation of self-healing hydrogel toward improving electromagnetic interference shielding and energy efficiency

**DOI:** 10.1038/s41598-021-95683-3

**Published:** 2021-08-09

**Authors:** Reza Peymanfar, Elnaz Selseleh-Zakerin, Ali Ahmadi, Ardeshir Saeidi, Seyed Hassan Tavassoli

**Affiliations:** 1 Laser and Plasma Research Institute, Shahid Beheshti University, G. C., Evin, 19839 Tehran, Iran; 2 Department of Polymer Engineering, Science and Research Branch, Islamic Azad University, Tehran, Iran; 3 Department of Chemical Engineering, Energy Institute of Higher Education, Saveh, Iran

**Keywords:** Materials chemistry, Electronic materials, Optical materials, Soft materials, Composites, Gels and hydrogels, Electronic properties and materials, Two-dimensional materials, Energy science and technology

## Abstract

In this study, a self-healing hydrogel was prepared that is transparent to visible (Vis) light while absorbing ultraviolet (UV), infrared (IR), and microwave. The optothermal features of the hydrogel were explored by monitoring temperature using an IR thermometer under an IR source. The hydrogel was synthesized using sodium tetraborate decahydrate (borax) and polyvinyl alcohol (PVA) as raw materials based on a facile thermal route. More significantly, graphene oxide (GO) and graphite-like carbon nitride (g-C_3_N_4_) nanostructures as well as carbon microsphere (CMS) were applied as guests to more dissect their influence on the microwave and optical characteristics. The morphology of the fillers was evaluated using field emission scanning electron microscopy (FE-SEM). Fourier transform infrared (FTIR) attested that the chemical functional groups of the hydrogel have been formed and the result of diffuse reflection spectroscopy (DRS) confirmed that the hydrogel absorbs UV while is transparent in Vis light. The achieved result implied that the hydrogel acts as an essential IR absorber due to its functional groups desirable for energy efficiency and harvesting. Interestingly, the achieved results have testified that the self-healing hydrogels had the proper self-healing efficiency and self-healing time. Eventually, microwave absorbing properties and shielding efficiency of the hydrogel, hydrogel/GO, g-C_3_N_4_, or CMS were investigated, demonstrating the salient microwave characteristics, originated from the established ionic conductive networks and dipole polarizations. The efficient bandwidth of the hydrogel was as wide as 3.5 GHz with a thickness of 0.65 mm meanwhile its maximum reflection loss was 75.10 dB at 14.50 GHz with 4.55 mm in thickness. Particularly, the hydrogel illustrated total shielding efficiency (SE_T_) > 10 dB from 1.19 to 18 and > 20 dB from 4.37 to 18 GHz with 10.00 mm in thickness. The results open new windows toward improving the shielding and energy efficiency using practical ways.

## Introduction

Recently, artificial intelligence has been the hotspot, leading to the revolution in the strategy of architecting the technologies in the present era. Smart devices are an inseparable part of the body of artificial intelligence. The smart devices are constructed from the high-tech subsets, needing technologies using the high frequencies to transport data^1^. Thus, electronic devices applying high frequencies have been developed toward facilitating our inescapable mechanical life. The charge circuits in electronic boards can establish the secondary fields and electromagnetic leakages which can be hazardous for any living species. On the other hand, the wireless devices employing electromagnetic waves have augmented our interactions by electromagnetic pollution. Thereby, to avoid receiving electromagnetic pollution by humans, microwave absorbing and shielding materials have been tailored. The permittivity and permeability of the absorbing or shielding structures play vital roles, paving the way for the microwave attenuation, as given by the transmission line theory^2–12^. Therefore, diverse structures have been applied to provide these features. Among them, the carbonaceous structures have been extensively used due to their significant polarization and conductive loss features. The achieved results manifest that the morphology of the aforementioned structures plays a vital role in regulating their microwave absorbing and shielding characteristics. The carbon nanotube, fiber, wire, sphere, graphene, flake, graphene foam, carbide, net, as well as other carbonaceous structures derived from the pyrolysis of the metal–organic framework (MOF)s and biomass-derived materials have attracted a great deal of attention in recent years ^13–22^. On the other hand, the absorbing matrix is the crucial parameter tuning the microwave characteristics. Paraffin wax as a conventional polymeric medium was used to investigate the microwave characteristics of the fabricated structures while its composites suffer from the proper mechanical properties. Other matrices including polystyrene, polyvinylidene fluoride, polymethylmethacrylate, silicone rubber, and so on have been widely used to strengthen mechanical properties of the shielding or absorbing structures and reinforce their microwave features^19, 20, 23–25^. These media promote the practical applications of the microwave absorbing and shielding composites in environmental, industrial, and military fields. Recent works have illustrated that the promising scenarios have been developed providing the novel perspective of microwave absorbers. Cao et al. have fabricated liquid crystalline elastomer and investigated its microwave characteristics^26^. Ionic conductive gels, which were transparent in visible light at room temperature, were architected by Fang et al. using phosphoric acid and PVA, demonstrating the significant microwave absorbing characteristics^27^. Recently, borax-crosslinked PVA hydrogel system with three-dimensional (3D) hierarchical network structures has demonstrated fascinating tissue-like, moldable, viscoelastic, biodegradable, flexible, tunable solvent content, biocompatible, mechanical, and lightweight properties. Noticeably, the characteristics of the aforementioned self-healing hydrogel were promoted by diverse fillers comprising polyaniline, carbon nanotube, polypyrrole, nanocellulose, etc. toward improving its intelligent applications in new-generation supercapacitors with smart functions, wearable and portable electronics, flexible and even self-reparative energy storage/conversion devices, soft bioelectronics and biosensors, electro-stimulated drug release devices and electronic skin, as well as bioelectrodes and strain sensors with excellent electrochemical performances^28–33^. Among them, cellulose-based structures were extensively applied in modern technologies including flexible/transparent substrates, batteries, super capacitors, electroactive materials in flexible sensors, and triboelectric nanogenerators due to their outstanding biocompatibility, low-cost, processability, biodegradability, and good mechanical flexibility. Particularly, this phenomenon is mainly originated from the potential of this type of material to modulate the chemical, structural, dielectric, and optical properties^34^. Thus, diverse approaches have been employed to modify the surface, defect, interface, polarizability, and chemical structure of nanocellulosic precursors for advanced materials^35^.

As known, the global warming has been the main challenge, exciting the global concern about the climate change. Improving the energy efficiency is the dominant way to prevent the global warming. In order to overcome the issue, the building materials have been developed based on their climate^36^.

Interestingly, various methods have been applied to reinforce the building materials against the electromagnetic waves^36^. It should be noted that the conventional electromagnetic shielding (ES) materials benefit from the impedance mismatching elevating the reflection at their interface. Thus, ES structures were architected based on the absorption, diminishing secondary hazards. Noteworthy, not only the large number of hydroxyl groups existing in the hydrogel are desirable for energy and ES efficiency, by absorbing IR waves and dipole polarization, but also by establishing hydrogen bonds with hydroxyl and amine groups related to CMS, GO, and g-C_3_N_4_ act as dispersing agents generating uniform composites. More significantly, the malleability of the hydrogel composites on one hand develop their practical applications as interlayer filler of laminated glass or double glazed windows on the other hand the self-healing feature of the 3D scaffolds reinforce their optical characteristics. Particularly, the ionic conductivity of the tailored hydrogels plays the key role, amplifying their microwave characteristics. In this study, a novel approach has been presented that not only diminishes energy consumption in the tropical regions but also enhances the ES efficiency based on absorption. A transparent self-healing hydrogel was fabricated through a facile route using borax as a raw material which can be used in our windows elevating energy and shielding efficiency.

## Experimental

### Materials

D-glucose (97.5–102.0%), sulfuric acid (95.0–97.0%), hydrochloric acid fuming (37.0%), and hydrogen peroxide (30.0%) were purchased from Merck meanwhile urea (98.0%), potassium permanganate (99.3%), sodium nitrate (99.0%), and borax 99.0–103.0%) were supplied from Samchun Chemicals. Moreover, PVA and graphite nanoparticles were obtained from Japan Vam & Poval Co. (POVAL™ JP-24) and US Research Nanomaterials, Inc. (C, 400 nm–1.2 µm, 99.9%), respectively.

### Experimental steps

#### Preparation of the fillers

The g-C_3_N_4_, GO, and CMS as the fillers were prepared using the reported literature^23, 37–39^.

#### Fabrication of self-healing structures

**Figure 1:**
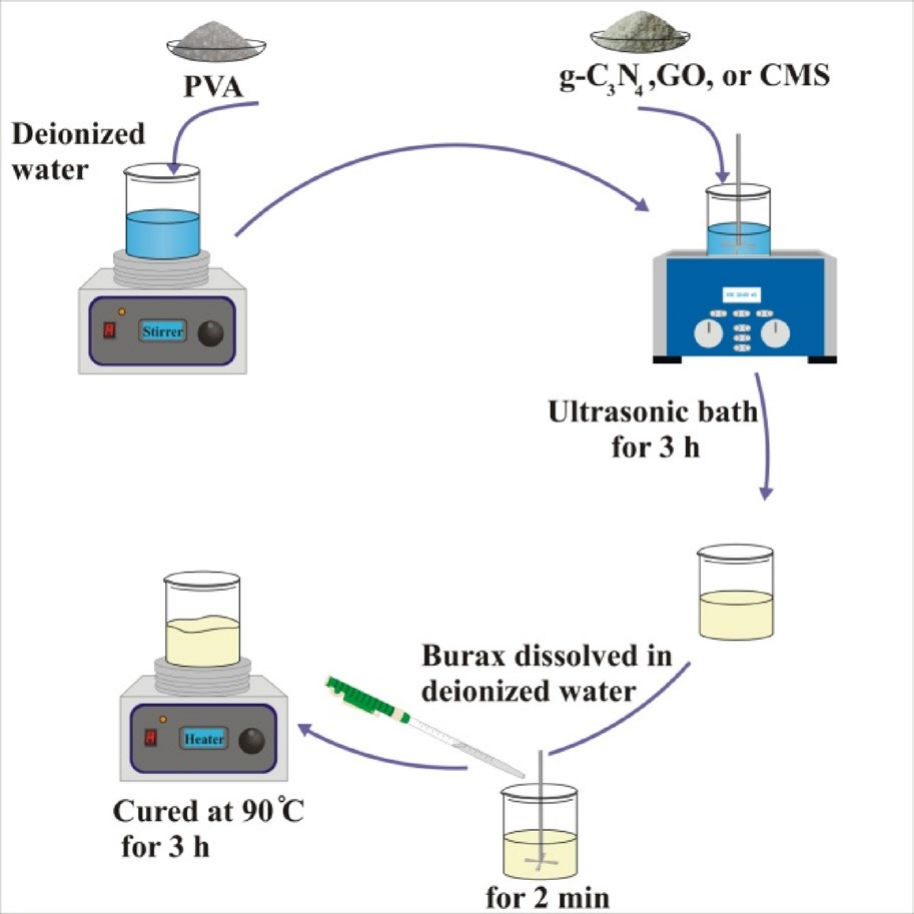
A schematic illustration of the synthetic procedure applied to prepare self-healing hydrogels.

PVA 5 wt% was dissolved in deionized water and the g-C_3_N_4_, GO, and CMS (guest/borax + PVA = 2 wt%) were separately suspended in the solution, following that the aforementioned suspension was simultaneously sonicated and stirred by an over head stirrer for 30 min. Afterwards, the borax (borax/PVA = 20 wt%) was dissolved in deionized water and added to the solution. The self-healing composites as novel microwave absorbers were synthesized by keeping the samples at 90 °C for 3 h. A self-healing hydrogel was prepared without any filler to compare the results, based on the presented procedures. Figure 1 depicts a schematic illustration of the synthetic procedure applied to prepare self-healing hydrogels.

### Characterization

The microwave characteristics were estimated by Agilent technology instrument (E8364A). The optothermal characteristics were examined by an IR source and monitored by an IR thermometer Lutron TM-958. The chemical species were identified using Shimadzu 8400 as well as Shimadzu MPC-2200 evaluated optical performance of the hydrogel at λ = 200–800 nm. MIRA3TESCAN-XMU revealed the morphology of guests. Stress–strain behavior of the samples was investigated by MCR 502 rheometer from Anton Paar.

## Results and discussions

### Morphology

**Figure 2:**
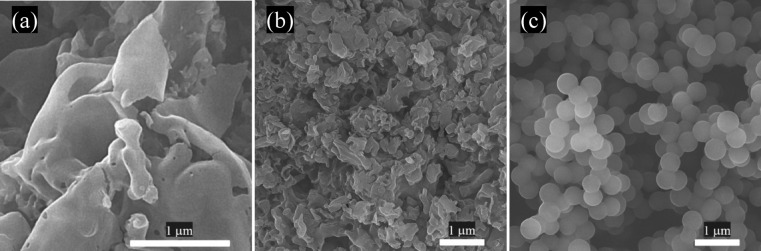
FESEM micrographs of GO (**a**), g-C_3_N_4_ (**b**), and CMS (**c**).

Figure 2 illustrates FESEM micrographs of the guests comprising GO, g-C_3_N_4_, and CMS. As indicated, all of the fillers have a uniform and smooth morphology. It can be seen that the self-assembly scenario of D-glucose along the hydrothermal process led to the formation of CMS with an average diameter of 600 nm^20^. More interestingly, 2D nanostructures with proper exfoliation and smooth surface of GO and g-C_3_N_4_ have been synthesized. It is well known that the more surface area to volume ratio augments the interfacial interactions in heterogeneous structures, desirable for relaxation loss mechanism^24, 39^.

### Chemical species

**Figure 3:**
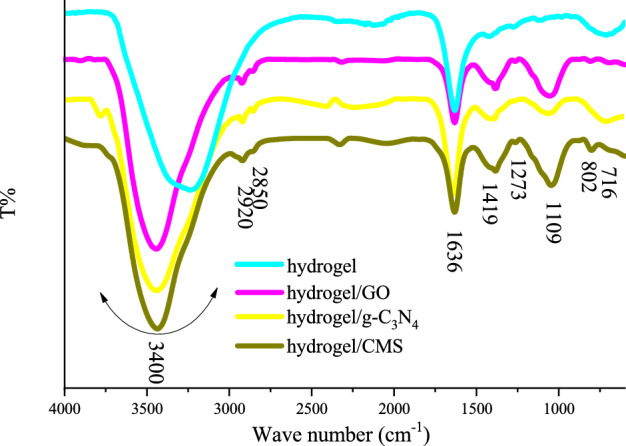
FTIR spectra of the samples.

Figure 3 presents FTIR spectrum of the samples. The assigned peak at 3400 and 1636 cm^−1^ are respectively ascribed to the stretching and bending vibrations of the hydroxyl functional groups existing in the hydrogel meanwhile the peaks at 2920 and 2850, and 802 cm^−1^ are related to the symmetric and asymmetric stretching vibrations as well as out-of-plane bending vibrations of C–H functional groups^14, 18, 31, 33, 40, 41^. Besides, the observed peaks at 1419, 1273, 1109, and bump at 716 cm^−1^ are associated with the stretching vibrations of B–O and C–O derivates including B–O–C, B–O–H, C–O–H, and C–O–B, as well as deformation vibration of O–B–O in the borate networks^42–46^. It can be seen that some of the peaks related to the conjugated structures have overlapped by the characteristics peaks of hydrogel^14, 20, 39^.

### Optical performance

**Figure 4:**
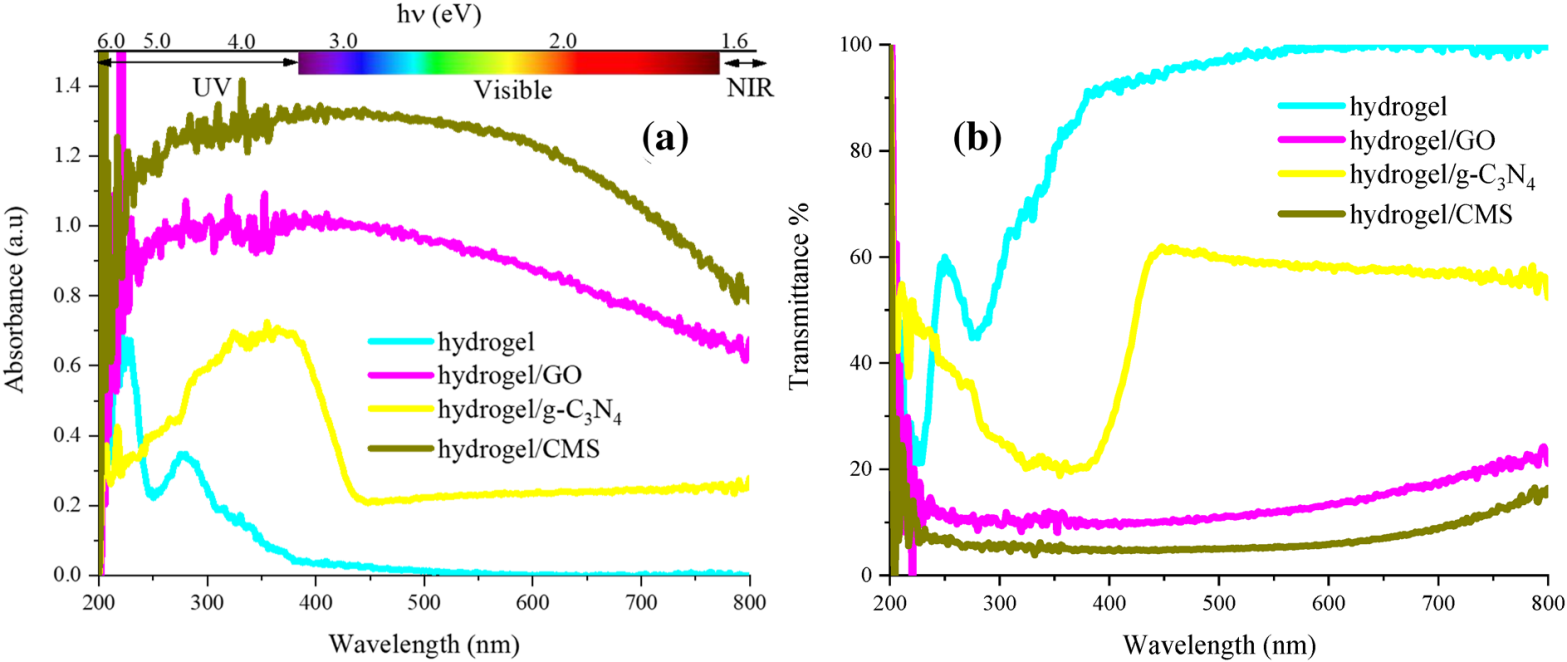
The light absorption (**a**) and transmittance % at λ = 200–800 nm (**b**) of the samples.

The optical performance of the hydrogel, hydrogel/GO, hydrogel/g-C_3_N_4_, and hydrogel/CMS were investigated from λ = 200 to 800 nm displayed in Figs. 4 and S1. The light absorption is mainly originated by the charge transmissions from the valance band to the conduction band. Evidently, the fabricated hydrogel is almost transparent in the Vis light meanwhile it can significantly absorb the UV light, confirmed by its energy band gap^25^. Particularly, the sample absorbs more than 80% of the UV light, implying the importance of the presented research in the practical application against UV light. The n → π* transitions could be a dominant parameter to produce the observed UV light absorption of the self-healing hydrogel. The displayed photographs refer to the visible transparency of the pure hydrogel meanwhile the loaded structures diminished the transparency of the fabricated structures (Fig. 4). The energy band gaps were evaluated based on Kubelka–Munk theory, given by the following equation: (α*h*ν)^2^  = *h*ν − *Eg*, α = *− *1/*t lnT*, and *T* = *10*^*−*A^, where ν, A, t, h, T, α, and E_g_ refer to the frequency, absorbance, thickness, Planck constant, transmittance, absorption coefficient, and energy gap. The achieved results clarified that the energy band gaps of hydrogel, hydrogel/GO, hydrogel/g-C_3_N_4_, and hydrogel/CMS were 3.90, 1.59, 2.92, and 1.44 eV, respectively. It is found that the inserted structures have regulated the distance between the highest occupied molecular orbital (HOMO) and lowest unoccupied molecular orbital (LUMO) of the fabricated structures, mainly generated from their intrinsic characteristics^20, 39^.

### Self-healing property and mechanical performance

**Figure 5:**
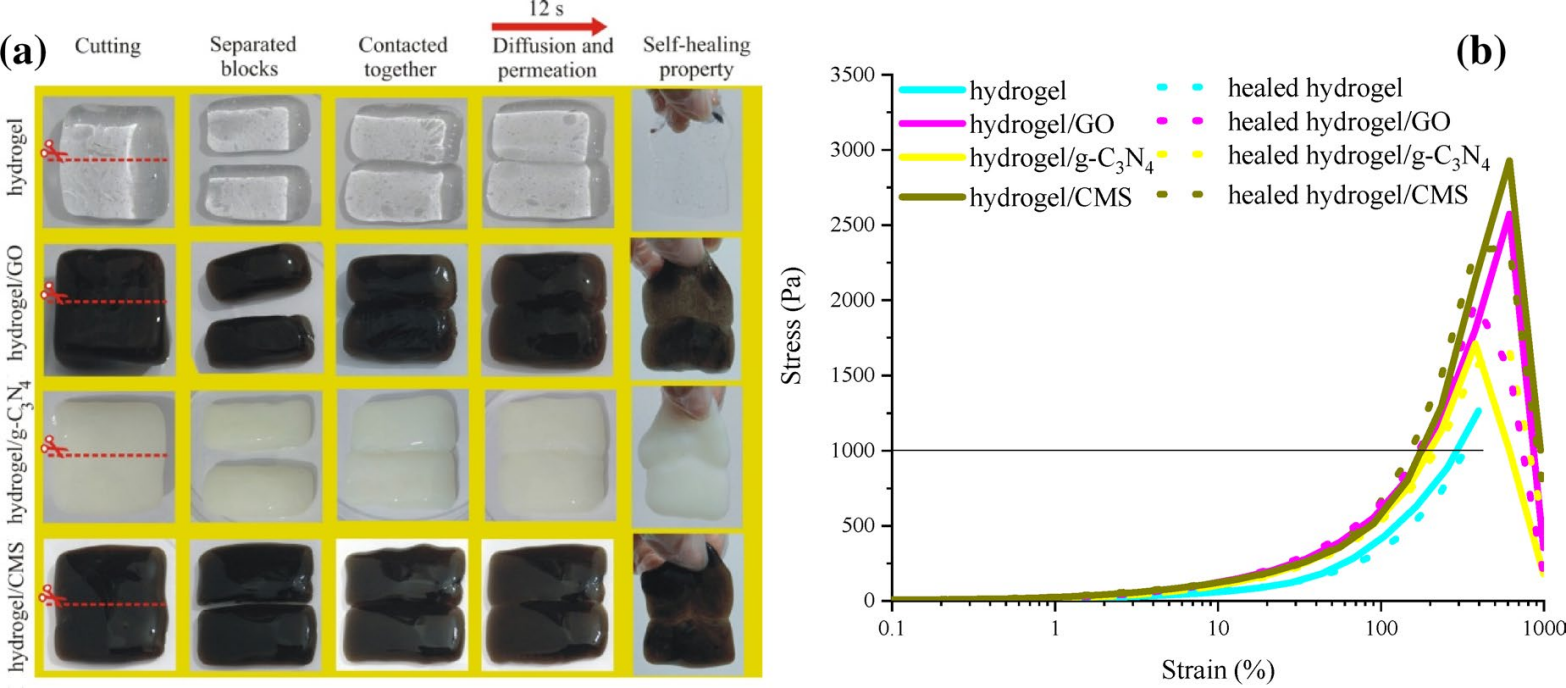
Self-healing procedure (**a**) and stress–strain curve (**b**) of the hydrogels.

Figure 5 depicts the self-healing procedure and stress–strain curve of the self-healing hydrogels. The fabricated self-healing hydrogels were cut into two equal parts. The separated blocks were contacted together at fractured zone without external stress or additional stimuli for 12 s at room temperature. Noticeably, the self-healing hydrogels were wholly merged. It is noteworthy that the self-healing time of the tailored self-healing hydrogels is so lower than that of polyacrylic acid-based self-healing structures^40, 41, 47, 48^. This spontaneous phenomenon is originated from diffusion and permeation at fractured interfaces, generated from the molecular dynamics reviving inter- and intramolecular hydrogen bonds, healing cross-links, and leading to more and more entanglements of the polymers^28, 29, 31–33^. Hydroxyl functional groups are mainly associated with PVA, CMS, GO, and water meanwhile amine groups come from g-C_3_N_4_, producing hydrogen bonds. More importantly, the established tetrahedron B(OH)_4_^¯^ acts as cross-linking agent. Tensile strength (F) and self-healing efficiency (η_F_ values of the self-healing hydrogels were summarized in Table 1 where η_F_ is evaluated by the following equation η_F_% = (F_healed_/F_original_)100^32, 33^. As revealed, at a stress of 1000 Pa the elongation of samples was ordered as hydrogel > hydrogel/g-C_3_N_4_ > hydrogel/GO > hydrogel/CMS meanwhile the tensile strengths were hydrogel/CMS > hydrogel/GO > hydrogel/g-C_3_N_4_ > hydrogel, derived from the emerged chemical interactions into the self-healing media.

**Table 1:** F and self-healing parameter of the samples.

Sample	F_original_ (Pa)	F_healed_ (Pa)	η_F_ (%)
Hydrogel	1259.5	1114.7	88.5
Hydrogel/GO	2567.5	1981.1	77.2
Hydrogel/g-C_3_N_4_	1703.8	1662.0	97.5
Hydrogel/CMS	2922.7	2342.4	80.1

### Optothermal features

**Figure 6:**
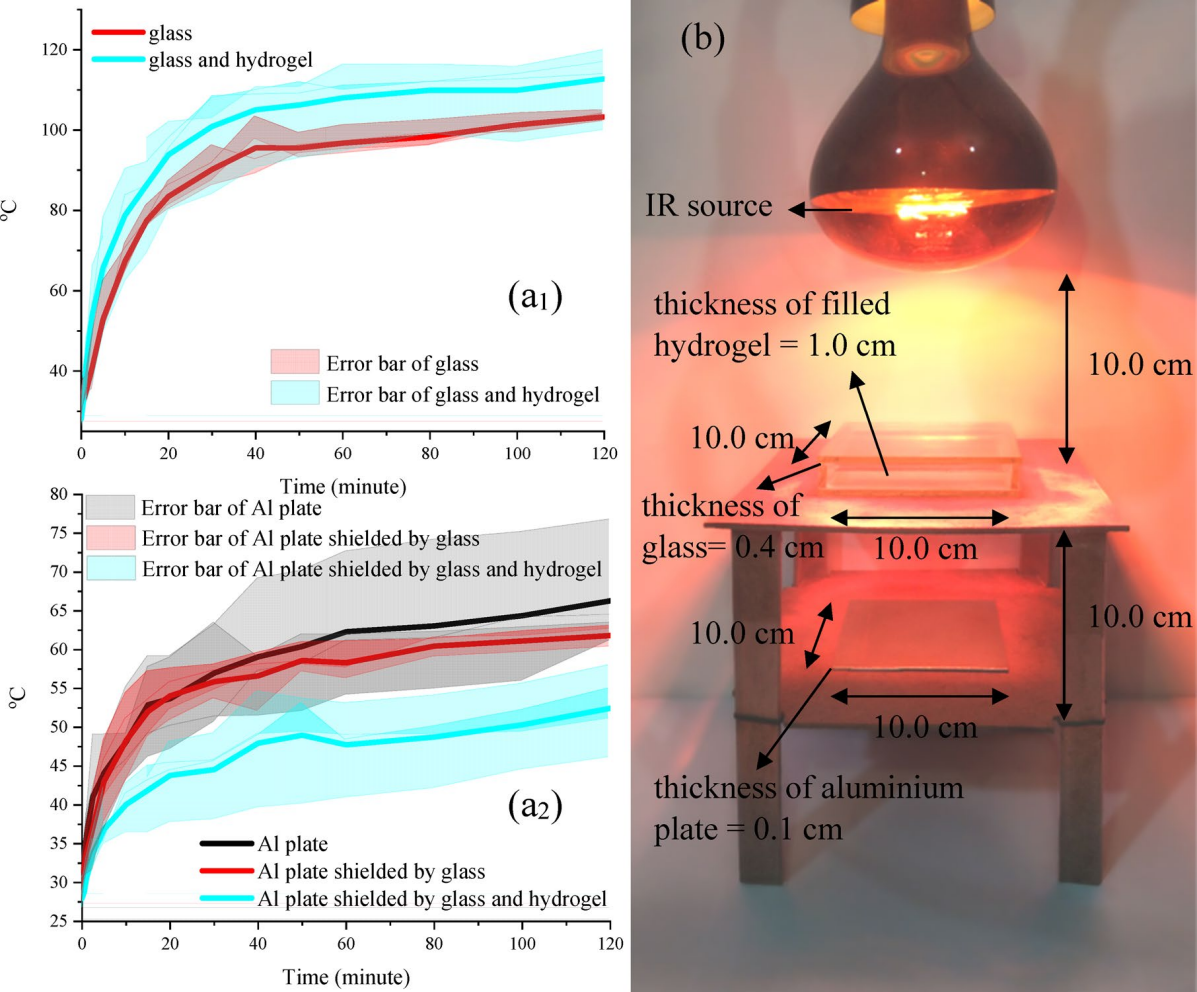
IR light absorption (**a**_**1**_**,**_**2**_) and optothermal setup (**b**).

The abundant portion of the sunlight is IR (about 50%), warming the earth along a day but it is improper to the tropical region. This phenomenon not only augments energy consumption due to keeping cool our buildings but also the used energy enhances the greenhouse effect creating secondary damages. Figure 6 exhibits IR light absorption of the fabricated hydrogel and the architected setup used to investigate the optothermal features. As indicated, a setup was architected to investigate the practical application of the hydrogel. Briefly, a cube was constructed using commercial glass with 0.4 cm thickness and 10.0 × 10.0 × 1.8 cm dimensions and then it was filled by the hydrogel. Afterwards, the fabricated cube was placed between the IR source and an aluminium plate (10.0 × 10.0 × 0.1 cm dimensions). Next, the temperature of the filled cube and aluminium plate under the IR source was monitored by an IR thermometer. Noteworthy, warming the glass and aluminium plate without the shielder were evaluated to more dissect and compare the results. It can be seen that the filled cube is warmed faster and more than the bare glass cube. On the other hand, the achieved results attested that the aluminium plate behind the filled glass cube was colder than the aluminium plate without shielder or with bare glass cube shielder. The obtained results manifest that the chemical structure of transparent self-healing hydrogel acts as an IR absorber, confining penetration of the incident waves. Therefore, the designed hydrogel can be applied as a novel structure in tropical regions keeping cool the buildings, desirable for improving energy efficiency.

### Microwave absorbing and shielding features

**Figure 7:**
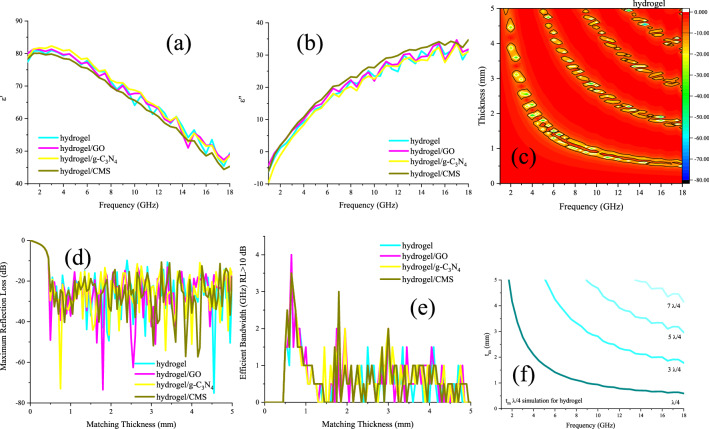
ε′ (**a**), ε″ (**b**), microwave absorption (**c**), maximum reflection loss *versus* matching thickness (**d**), efficient bandwidth (RL > 10 dB) *versus* matching thickness (**e**), and simulation of matching thickness (**f**) for the samples from 1 to 18 GHz.

**Figure 8:**
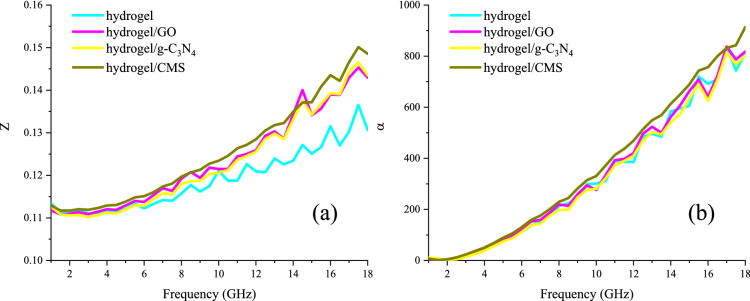
Z (**a**) and α (**b**) of the microwave absorbing structures.

Figures 7 and S2 presents the real part of permittivity (ε′), imaginary part of permittivity (ε″), microwave absorption, maximum reflection loss *versus* matching thickness, efficient bandwidth (RL > 10 dB) *versus* matching thickness, and simulation of matching thickness for the samples from 1 to 18 GHz. The real part was originated from the storage of the waves meanwhile the imaginary part came from the dissipation of them. GO, g-C_3_N_4_, and CMS were employed as guests to more examine their influence on the microwave features of the hydrogel. Evidently, by enhancing the frequency the real parts of the samples are descended nevertheless their imaginary parts are ascended. The permittivity of the hydrogel is mainly generated from its dipole polarization, electron hopping, and established ionic conductive network. Microwave absorptions of the samples were evaluated by the transmission line theory^49–51^. As given by the results, the efficient bandwidth of the hydrogel was as wide as 3.5 GHz with only a thickness of 0.65 mm meanwhile its maximum reflection loss was 75.10 dB at 14.50 GHz with 4.55 mm in thickness. It can be seen that the quarter wavelength mechanism is the dominant parameter bringing microwave absorption of the samples, defined by the attenuated waves by the reversal waves when they are 180° out of phase of incident waves and the thickness of the absorber is an odd numeral of the λ/4 of penetrated waves^52^. Noteworthy, the type of fillers modulates the maximum reflection loss and efficient bandwidth, derived from their unique characteristics including conductive loss as well as interfacial and dipole polarization. However, the obtained results manifest that the hydrogel performs the essential duty for the microwave absorbing features. Impedance matching (Z) is the crucial factor that has a clear trade-off between the propagation of the incident waves in the absorbing medium (Z = 1). As revealed, not all samples have the proper impedance matching, but loading the fillers augmented Z. The attenuation constant (α) implies the potential of an absorber for energy conversion that has a clear compromise with imaginary parts of the permittivity and permeability^53^. Figure 8 displays Z and α of the microwave absorbing structures.

**Figure 9:**
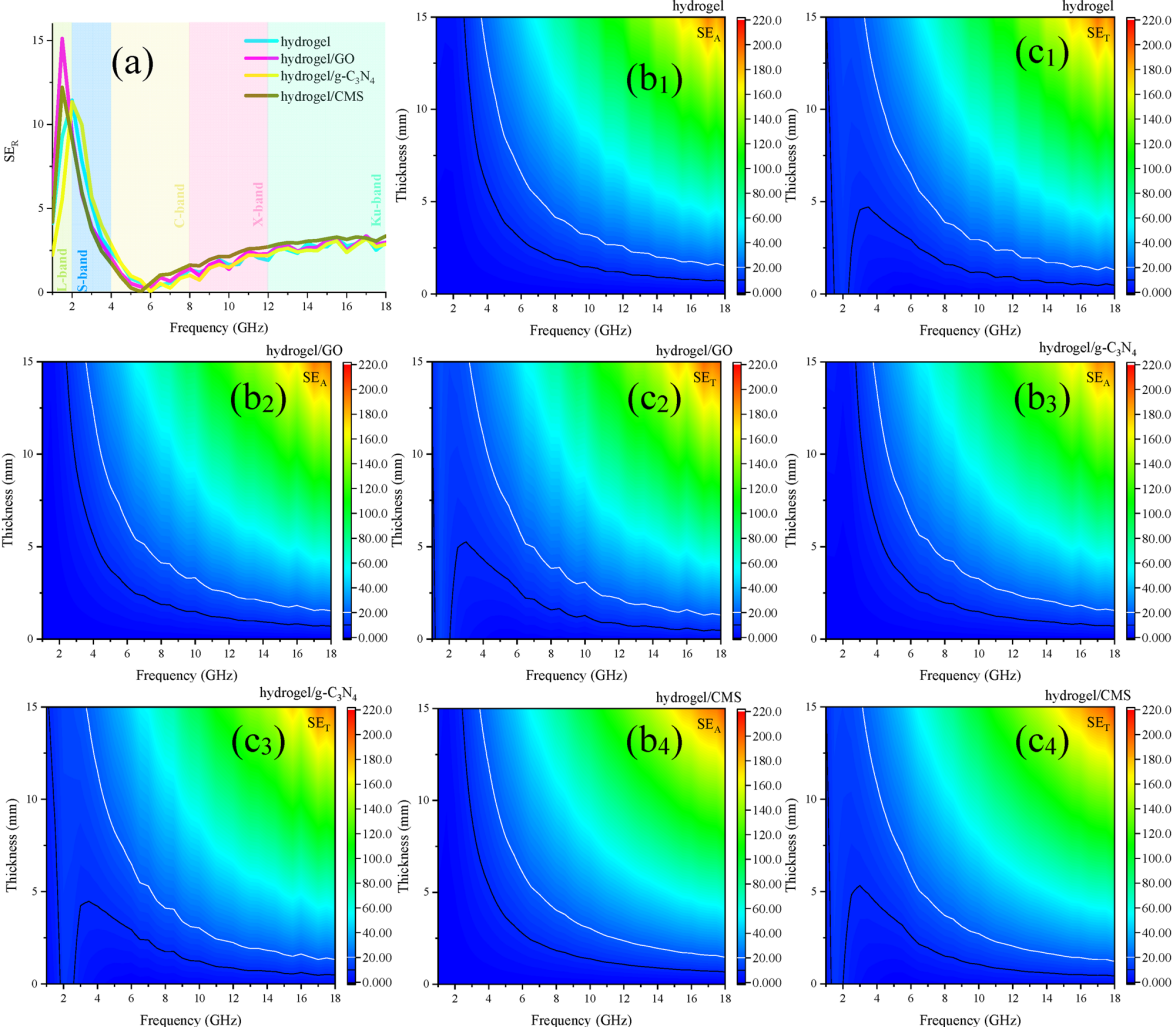
SE_R_ (**a**), SE_A_ (**b**_**1–4**_), and SE_T_ (**c**_**1–4**_) of the hydrogel containing structures.

SE_T_ is obtained from the sum of the shielding efficiency of absorbance (SE_A_) and reflectance (SE_R_)^54^. Obviously, SE_R_ is independent of the thickness as confirmed by its equation. All of the samples exhibited considerable SE_T_, established by their SE_A_. Interestingly, absorbance plays a vital role in paving the way for the shielding property of the samples containing hydrogel. This phenomenon diminishes the secondary pollution, produced by the reflectance at the interface of the shielding structures. Particularly, the hydrogel illustrated total shielding efficiency SE_T_ > 10 dB from 1.19 to 18 and > 20 dB from 4.37 to 18 GHz with 10.00 mm in thickness. Figure 9 exposes SE_R_, SE_A_, and SE_T_ of the samples.

**Figure 10:**
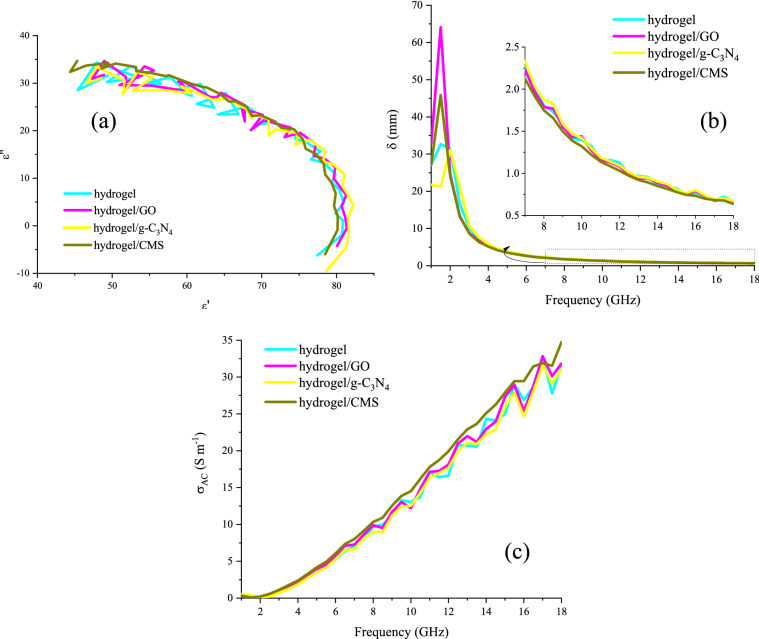
Cole–Cole plot (**a**), δ (**b**), and σ_AC_ (**c**) for the prepared samples.

**Figure 11:**
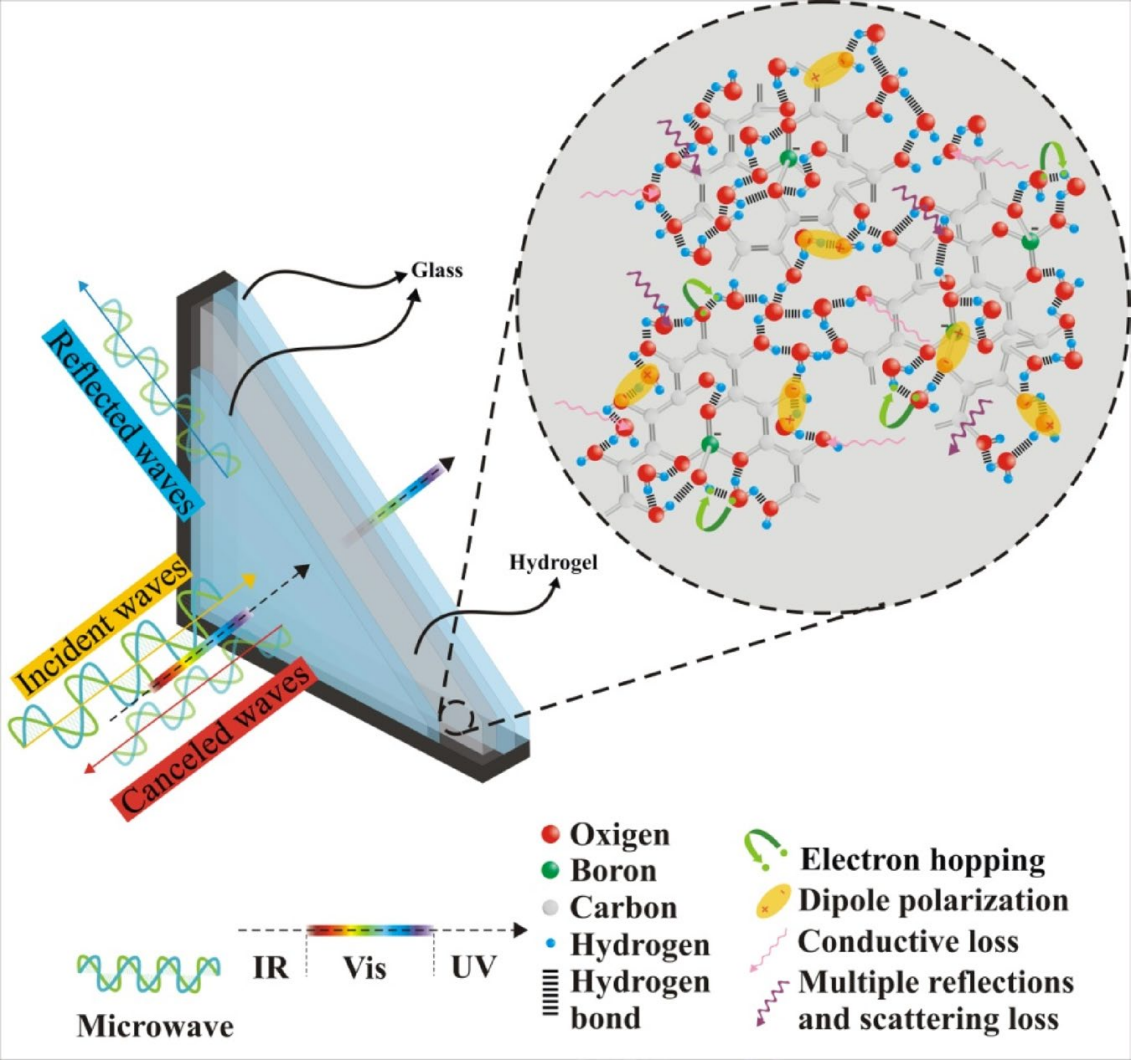
An illustrative diagram of the commanding mechanisms in the hydrogel.

Based on the Debye relaxation theory, each emerged semicircle, produced by drawing ε′ *versus* ε″ refers to one relaxation process, known as Cole–Cole plot^55^. The plots attest that all of the samples follow the distinct pattern confirming that only one type of polarization exists in the absorbing media, generated from the dipole polarization and charge transfer in the self-healing hydrogel. Skin depth (δ) is the dominant factor regulating SE of structures^56–58^. This factor is modulated by alternative conductivity (σ_AC_) which is tuned by ε″^59^. Thus, the imaginary part of the permittivity plays a crucial role in modulating SE of the shielding materials. There are n → σ* transitions from the lone pair electrons of oxygen and σ* of hydrogen coupled to the oxygen (hydrogen bonds). As indicated, σ_AC_ is enhanced by elevating the frequency implying that by increasing the energy the charge transitions, originated from the ionic conductivity and relaxation loss are promoted. Figure 10 depicts the Cole–Cole plot, δ, and σ_AC_ for the tailored specimens. Figure 11 exhibits an illustrative diagram of the commanding mechanisms in the hydrogel. The above-mentioned equations were presented in the supporting information.

## Conclusion

In this research, a self-healing hydrogel was fabricated which was transparent in Vis light while absorbing IR, UV, and microwave. This ability opens new windows toward reinforcing our building against harmful microwave irradiations by using the hydrogel in windows. Interestingly, the hydrogel illustrated considerable IR absorption attesting to its ability to apply in the tropics, keeping our buildings cool through its use in windows, for example. Diverse fillers were applied to more dissect the microwave absorbing and shielding characteristics. It should be noted that the obtained shielding results of the samples were based on the absorption diminishing the secondary pollution established by the reflection at the shielding interface. It can be seen that the nanofillers and CMSs have no significant influence on microwave characteristics. The achieved results manifest that the dipole polarization, electron hopping, and produced ionic conductive network are the pioneer mechanisms bringing microwave properties of the structures.

## Supplementary Information

Supplementary Information.
